# How scan parameter choice affects deep learning-based coronary artery disease assessment from computed tomography

**DOI:** 10.1038/s41598-023-29347-9

**Published:** 2023-02-13

**Authors:** Felix Denzinger, Michael Wels, Katharina Breininger, Oliver Taubmann, Alexander Mühlberg, Thomas Allmendinger, Mehmet A. Gülsün, Max Schöbinger, Florian André, Sebastian J. Buss, Johannes Görich, Michael Sühling, Andreas Maier

**Affiliations:** 1 Pattern Recognition Lab, Friedrich-Alexander-Universität Erlangen-Nürnberg, Erlangen, Germany; 2 Computed Tomography, Siemens Healthcare GmbH, Forchheim, Germany; 3 Department Artificial Intelligence in Biomedical Engineering, Friedrich-Alexander-Universität Erlangen-Nürnberg, Erlangen, Germany; 4 Das Radiologische Zentrum-Radiology Center, Sinsheim-Eberbach-Erbach-Walldorf-Heidelberg, Germany

**Keywords:** Cardiovascular diseases, Cardiovascular biology, Computer science

## Abstract

Recently, algorithms capable of assessing the severity of Coronary Artery Disease (CAD) in form of the Coronary Artery Disease-Reporting and Data System (CAD-RADS) grade from Coronary Computed Tomography Angiography (CCTA) scans using Deep Learning (DL) were proposed. Before considering to apply these algorithms in clinical practice, their robustness regarding different commonly used Computed Tomography (CT)-specific image formation parameters—including denoising strength, slab combination, and reconstruction kernel—needs to be evaluated. For this study, we reconstructed a data set of 500 patient CCTA scans under seven image formation parameter configurations. We select one default configuration and evaluate how varying individual parameters impacts the performance and stability of a typical algorithm for automated CAD assessment from CCTA. This algorithm consists of multiple preprocessing and a DL prediction step. We evaluate the influence of the parameter changes on the entire pipeline and additionally on only the DL step by propagating the centerline extraction results of the default configuration to all others. We consider the standard deviation of the CAD severity prediction grade difference between the default and variation configurations to assess the stability w.r.t. parameter changes. For the full pipeline we observe slight instability (± 0.226 CAD-RADS) for all variations. Predictions are more stable with centerlines propagated from the default to the variation configurations (± 0.122 CAD-RADS), especially for differing denoising strengths (± 0.046 CAD-RADS). However, stacking slabs with sharp boundaries instead of mixing slabs in overlapping regions (called true stack ± 0.313 CAD-RADS) and increasing the sharpness of the reconstruction kernel (± 0.150 CAD-RADS) leads to unstable predictions. Regarding the clinically relevant tasks of excluding CAD (called rule-out; AUC default 0.957, min 0.937) and excluding obstructive CAD (called hold-out; AUC default 0.971, min 0.964) the performance remains on a high level for all variations. Concluding, an influence of reconstruction parameters on the predictions is observed. Especially, scans reconstructed with the true stack parameter need to be treated with caution when using a DL-based method. Also, reconstruction kernels which are underrepresented in the training data increase the prediction uncertainty.

## Introduction

CAD continues to be one of the most severe human diseases with a frequent deadly outcome^1^. Commonly, its root cause is inflammation of perivascular tissue, leading to atherosclerosis, i.e., aggregation of plaque deposits within the vessel walls. These deposits may cause a narrowing of the vessel—so-called stenosis—which may lead to a malperfusion of the heart muscle and therefore cardiac ischemia and a higher risk of acute cardiac death^2^. Also, these plaques can rupture, leading to thrombus formation and thus potentially causing stroke or myocardial infarction. A non-invasive modality capable of assessing the severeness of CAD is CCTA. Contrast agent injected during a Computed Tomography (CT) acquisition enhances the vessels, allowing stenotic lesions to be detected. Commonly, the severeness of CAD, as manifested in CCTA scans, is assessed using the CAD-RADS score^3^). The most severe stenotic lesion within a patient’s coronary tree is the main contributor to this score. However, also the location of this culprit lesion and some qualitative aspects are considered when determining this score. Relevant subgroups within the six grades of the CAD-RADS score are CAD-RADS 0, referring to no CAD being present, CAD-RADS 1–2, referring to a non-obstructive CAD without need for further (invasive) assessment and CAD-RADS 3–5 being assigned to patients who should undergo immediate further assessment. The resulting clinical questions are whether a patient has CAD or not (rule-out) and whether a patient has obstructive CAD or not (hold-out). In general, the CAD-RADS score is determined manually by a human reader grading the whole coronary artery tree. This procedure is time-consuming, and with the increasing workload radiologists need to cope with, interest in supporting algorithms is high.

We recently proposed such an algorithm^4^ which is Deep Learning (DL)-based and directly predicts the CAD-RADS score using a task-specific data representation and architecture design. A high-level overview of this method is displayed in Fig. 2. It consists of multiple steps: First, the heart is roughly isolated from the rest of the scan^5^. Then, centerlines of the coronary arteries are extracted from the CCTA volume^6^ and subdivided into sub-segments. Next, for each of these sub-segments a Multi Planar Reformatted (MPR) volume stack is extracted by interpolating planes orthogonal to each centerline point. Finally, from these MPR volumes, longitudinal views through the centerline are sliced for each respective sub-segment and individually fed into a shared 2D feature extraction Convolutional Neural Network (CNN). The resulting feature representation is used to predict a segment-wise stenosis degree label and global max-pooling of the representations is leveraged to predict the patient-wise CAD-RADS grade and the Agatston score binned according to Rumberger and Kaufman^7^ as additional auxiliary target.

This method reaches high performance on the task of regressing all six CAD-RADS grades as well as for the rule-out and hold-out task, with an average accuracy of 0.859 for the six class problem and an AUC of 0.942 and 0.950 for the rule-out and hold-out case, respectively.

Before we go into detail on our methodology in this paper, we want to sketch the bigger picture and discuss variances within the whole measurement system of a CCTA analysis. First, a patient, who exhibits different characteristics like weight, shape, disease state, position, etc., undergoes a CT scan. The resulting projection data is not only influenced by the patient’s characteristics but also by the type of scanner, the tube voltage, and the dosage of contrast agent applied. Next, the projection images are reconstructed, whereby the choice of reconstruction kernel, the amount of applied denoising, the heart phase for which the scan is reconstructed, the way neighboring slabs from different heart cycles are stacked together influence the appearance and content of the final volume. Finally, the resulting images are interpreted by a human or Artificial Intelligence (AI) reader. An experienced human reader might be able to disentangle the change in visual perception caused by different acquisition parameters from the actual biological information. However, an AI system, which may have only seen training samples from a subset of fixed scan and reconstruction parameters, is probably influenced by these different technical variations.

Examples for this are already described in literature and can be divided into analyses focusing on the impact of image formation parameter choice on classical Machine Learning (ML)^8–11^ on the one hand and on DL^12–14^ approaches on the other.

Wielpütz et al.^10^ examined the influence of the tube voltage selection and whether Filtered Back Projection (FBP) or Iterative Reconstruction (IR) is used for the volume reconstruction for the task of detecting artificial nodules in an ex vivo study. They found that there was no significant impact on the evaluated classical ML algorithm. In contrast, Berenguer et al.^8^, and Li et al.^9^ showed that Radiomic features (which include shape-based and first- and second-order statistics on a selected Region of Interest (ROI)) are often not reproducible if one of various scan parameters or the scanner type is varied^8^. Also, the performance of models based on these features may drop^9^. Moreover, Reiazi et al.^11^ confirmed that feature distributions vary for different scanner types. For classical ML algorithms, research to compensate differing image formation parameters exist based on statistical assumptions^15^ or technical fingerprints in control regions^16^.

For DL-based algorithms, analysis of the influence of the image formation parameter was mainly performed on the task of CT lung imaging. Li et al.^12^ demonstrated that the performance on the task of detecting nodules changes slightly when the tube voltage or the reconstruction type is varied in a phantom study. A comparable study was conducted by Blazis et al.^13^ with a commercially available AI-based system for nodule detection. They used raw data from 24 patients and evaluated 16 different reconstruction settings varying the kernel, denoising strength and reconstruction type. They found an impact of all parameters on the sensitivity of the examined system. Another paper published by Hoang Thi et al.^14^ evaluated whether reconstructions with both sharp and soft kernels should be included within training of an algorithm to segment lung nodules. They concluded that the performance is only transferable between kernel types if all options are included in the training step of the algorithm. Recently, the impact of acquisition and patient parameters on an AI-guided CAD assessment system was evaluated^17^. The underlying pipeline consists of ML-based centerline extraction and labeling, inner and outer wall segmentation and lesion detection and scoring systems. However, the final prediction of each step is double-checked by a human reader to prevent error propagation. They explore several different variations of acquisition and patient parameters including the scanner type, tube voltage, gating technique, several clinical parameters, etc.. Limitations of this work are that the individual subgroups differ in size and that the impact of a single parameter change on the system cannot be directly measured but needs to be statistically assessed over a large patient population. Furthermore, the influence of the variations on the AI components cannot be separated from the additional human reader.

**Figure 1:**
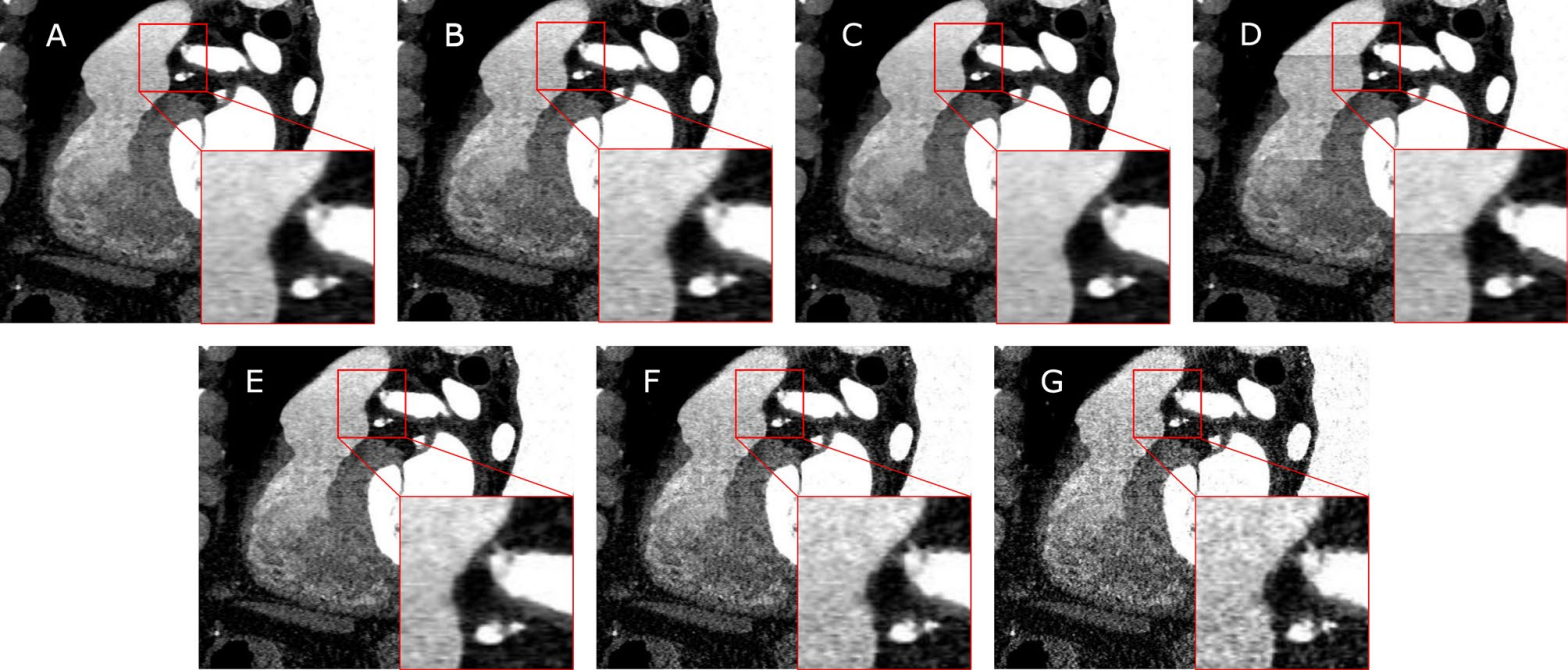
Sagittal views of a CCTA scan reconstructed with different parameters. The default configuration (**A**) reconstructed with an Advanced Modeled Iterative Reconstruction (ADMIRE) strength of 3, mixed stack and Bv36 kernel is varied by using: an ADMIRE strength of 2 (**B**) or 4 (**C**), using true stack (**D**) and utilizing a Bv40 (**E**), Bv44 (**F**) or Bv49 (**G**) reconstruction kernel.

After this brief overview of related work, we will define the scope of this work. The image formation parameter choice often differs for different clinical sites and personal preference. Therefore, when considering the clinical application of an AI-based CAD approach, it is crucial to evaluate how well this algorithm performs for differing reconstruction parameters. With this work, we aim to analyse the influence of a set of reconstruction parameters on our previously published CAD-RADS scoring system^4^. These parameters are defined in more detail in “Raw prediction changes” and include the ADMIRE strength, stacking and reconstruction kernel choice. Image impressions of these altered reconstruction parameters are depicted in Fig. 1. To systematically evaluate the influence of each of these parameters on our AI system, we leverage a collection of 500 raw data sets, and reconstruct all samples with one default configuration and single parameter variations. The CAD-RADS grading method consists of several preprocessing and a Neural Network (NN) prediction step. To be able to separate the variation changes’ impact on the NN step of the pipeline from the impact on the preprocessing, we evaluate the full pipeline and the pipeline with the centerlines propagated from the default configuration to all variations. Our contributions can be summarized as follows:

- To the best of our knowledge, we conduct the first evaluation of scan parameter dependency of a DL-based approach for automatic assessment of CCTA scans with paired data, i.e. the sole difference between the individual reconstructions being the parameter change.
- We separate the influence of the parameter changes on the preprocessing results from the change in image data.
- We provide guidance regarding which image formation parameters need to be treated with caution.

## Methods

All the methods in this study were performed in accordance with the Declaration of Helsinki.

### Data

Before going into detail about the data used in this study, the distribution of labels and reconstruction parameters, we want to define the parameter space we evaluate. An exemplary case for each parameter configuration is displayed in Fig. 1.

#### ADMIRE strength

A parameter that might influence the prediction and is sometimes altered in clinical practice is the number of iterations of the reconstruction algorithm. In this study, we use the ADMIRE algorithm^18^ to denoise already during reconstruction. Depending on the number of iterations, the algorithm reduces noise but also may introduce denoising artifacts. A popular choice in clinical practice is a ADMIRE strength of 3 (default) with variations to 2 or 4 depending on the image quality and personal reader preference. We therefore reconstructed our raw data with these three parameter choices.

#### Stacking

Mostly, CCTA projection images are acquired over multiple heart cycles as the field of view of the detector is usually not large enough to cover the whole heart in one rotation. Therefore, the patient table is moved along the superior-inferior axis during the acquisition. In addition, depending on the motion occurring between heart cycles, e.g. breathing motion, the projections for different z-positions may not match each other directly at their boundaries. Since there are usually overlapping regions for the patient positions, there are two possible strategies to cope with this: either the overlapping regions are merged using interpolation strategies (mixed stack, default), or only the information of a single position is preserved (true stack). As the first strategy may introduce artifacts when a lot of motion occurs between the heart cycles, physicians prefer the true stack option in these cases. However, it leads to sharp boundaries between the individual stacks, which do not necessarily impact human readers but may impact the performance of algorithms processing the volume (cf. Fig. 4).

#### Reconstruction kernel

Finally, another important parameter that is often changed in clinical practice is the reconstruction kernel. By adapting it, the sharpness of the edges can be increased at the cost of an increased noise level. Each vendor offers its own set of reconstruction kernels. For our experiments, we choose the Siemens Healthineers specific Bv36 kernel as the default, which is a medium sharp kernel specifically designed for the heart anatomy and therefore commonly used for CCTA scans. As we observed an increase in instability correlating with kernel sharpness during initial experiments we chose the increasingly sharp Bv40, Bv44, and Bv49 kernels as variations. With all of these variations, the volume content should be consistent while the appearance may change (cf. Fig. 1).

#### Data characteristics

We use two data collections in this study. Once, a data collection of 2596 reconstructed CT scans (data set A) as training set for the CAD-RADS scoring system. Additionally, we leverage a data collection containing raw CT data of 500 patients (data set B). Both data collections were collected at the same center with Siemens SOMATOM Force scanners. All samples in data collection A were reconstructed using the Bv36 reconstruction kernel with a slice thickness of 0.6 mm. Furthermore, the ADMIRE reconstruction algorithm was applied with a strength of 3. 55 cases were reconstructed using true stack and all others with mixed stacking. The CAD-RADS class frequency in the training set (A) is 370, 551, 828, 542, 281, 24 for CAD-RADS 0 to 5 respectively. For the raw data collection B 7 configurations (examples displayed in Fig. 1) were reconstructed for all 500 data samples: a default configuration (ADMIRE strength = 3; stacking = mixed; kernel = Bv36) varied by using an ADMIRE strength of 2 or 4, true stacking and a Bv40, Bv44 or Bv49 reconstruction kernel. Reconstruction was performed with ReconCT (version 15.0, Siemens Healthineers). For data set B the class distribution is more balanced with 73, 61, 81, 85, 146, 54 samples for each respective CAD-RADS grade.

### Algorithm

**Figure 2:**
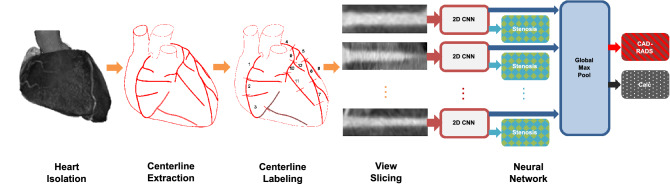
Overview of the used AI approach. First the heart is isolated from the CCTA scan using the algorithm proposed by Zheng et al.^5^. Then the coronary centerlines are extracted^6^. These are subdivided into up to 11 equally sized sub-segments^4^. For each sub-segment, longitudinal slices are interpolated orthogonal to the centerline and fed into a NN.

A high-level overview of the method evaluated in this work is depicted in Fig. 2. As this scientific publication focuses on the evaluation of scan parameter influences, we refer to the publication where the evaluated method was proposed^4^ for most details. Still, we want to mention some properties which have impact on the robustness analysis. From the CCTA scan data to the final prediction, multiple different algorithms are utilized. These include an algorithm for creating a rough segmentation of the heart^5^, extracting the centerlines^6^, and labeling them^4^. As each of the later steps depends on the preceding ones, differences are propagated through the whole pipeline, altering the final prediction. Centerline labeling does not depend on the image data but solely leverages the centerline coordinates. The last pipeline step is the data processing through a task-specific DL-based architecture. One forward pass of this architecture takes one longitudinal slice of each labeled coronary segment as an input. In order to include all information in the final prediction the concept of Test Time Augmentation (TTA) is leveraged by extracting these longitudinal slices at 16 equidistant angles around the centerline as rotation axis within a range of [0, $\pi$] from the volume. The average prediction over all angles is considered the final prediction for a single model. This is done to prevent the algorithm from missing information due to unfortunate angle choices. Also, the models of 25 training runs with different randomly chosen training and validation splits are ensembled to increase the method’s stability and performance. We encode the prediction in a multi-label format. As, if a patient belongs, e.g., to the CAD-RADS 3 category, he also fulfills the criteria of CAD-RADS 0-2 due to a gradual nature of the score (i.e., (1, 1, 1, 1, 0, 0) represents CAD-RADS 3). We consider the predicted cumulative probability of all classes as the raw output score. Due to class imbalance default thresholds of .5 between the raw predictions do not necessarily lead to optimal class predictions. To circumvent this problem we determine more optimal thresholds: we define them as the percentiles of the raw prediction histogram. The percentile values are defined by the class frequencies. E.g. if 73 cases belong to the CAD-RADS 0 and 61 to the CAD-RADS 1 class, the threshold between CAD-RADS 1 and CAD-RADS 2 is the 134th lowest prediction.

As we focus on having the best possible class separation for the robustness analysis, we calculated the thresholds on the test set predictions of the default configuration reconstructions. For the performance analysis we use the threshold invariant AUC metric.

### Evaluation

As the focus of this work is the evaluation of the stability of our CAD-RADS estimation approach, we choose to compare predictions of the default parameter configuration to the individual parameter variations. Since with the step of binning the network’s CAD-RADS predictions to distinct scores a lot of information is lost we choose to evaluate the difference in raw prediction scores. These also encode a kind of certainty regarding the prediction. To render the raw scores comparable for all individual CAD-RADS grades we rescale the predictions such that the value range between two thresholds always equals 1. As metrics, we evaluate whether the parameter change leads to a shift in the mean prediction and how much the standard deviation over all patients changed. Also, the number of cases where the parameter change leads to a different binned prediction is of interest, although outliers may influence it. Finally, the overall performance of the method regarding the hold-out and rule-out case may vary. Here, we decide to focus on the AUC as a threshold independent metric, also because the thresholds were defined on the test set. Also, to separate the influence of the parameter change on the NN component; we evaluate the influence of the deviations if we propagate the centerlines extracted from the default configuration to all others. Furthermore, as our approach relies on model ensembling, TTA, and a large training data cohort, which are all known factors to increase the robustness of DL-based models, we conduct additional experiments without model ensembling, without TTA, and with random subsets of only 10% (259 patients) or 20% (519 patients) of the training data.

### Ethical standards

The CT examinations were clinically indicated by the referring physicians and conducted in accordance with current clinical standards, guidelines, and recommendations. The study was performed in accordance with the Declaration of Helsinki and was approved by the local ethics committee (S-226/2016 and S-758/2018, Ethikkommission der Medizinischen Fakultät Heidelberg, Germany). Subjects included as of January 2019 gave informed consent in the scientific data analyses. For the retrospective analyses of the datasets acquired before January 2019, a waiver of consent was granted by the aforementioned ethics committee.

## Results

### Raw prediction changes

**Figure 3:**
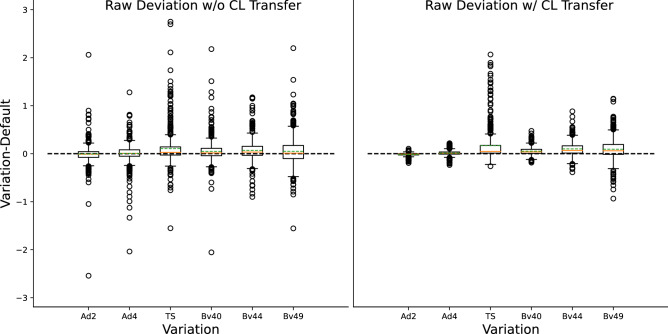
Boxplot of the rescaled raw prediction difference between the default configuration and all variation configurations (Ad2, Ad4 = ADMIRE strength of 2/4; TS = true stack; Bv40, Bv44, Bv49 = different reconstruction kernels), with and without centerline propagation.

**Figure 4:**
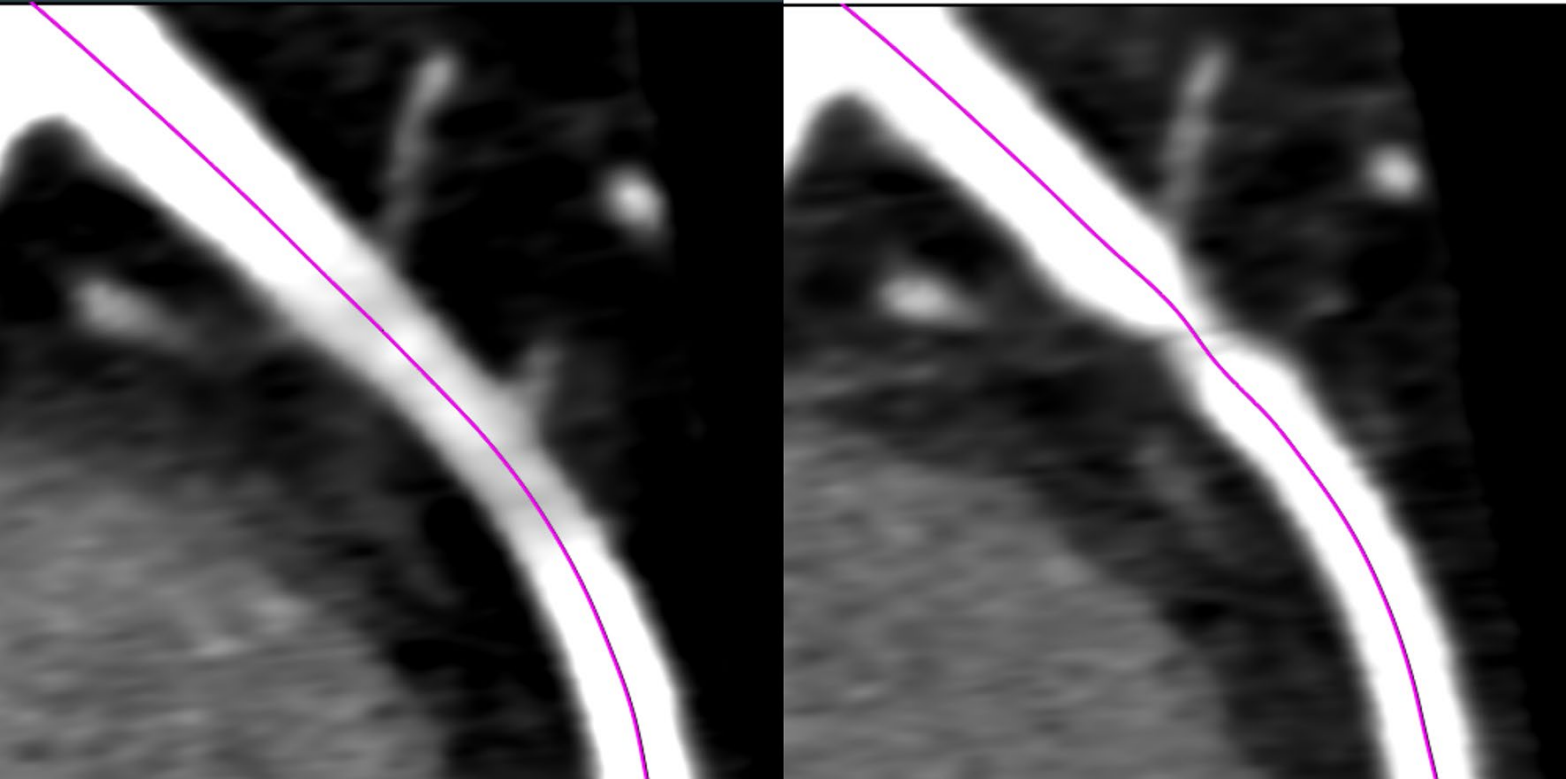
Curved Planar Reformatted (CPR) view of the Right Coronary Ascending (RCA) proximal segment for a reconstruction with mixed stacking (left) and true stacking (right) of the same raw data set. Due to the sharp slab boundaries the visual perception suggests a narrowing of the vessel.

First, we want to report the changes in raw predictions caused by varying the image formation parameters. Therefore, the prediction difference between the default and the respective variation for the whole data set is visualized as boxplot in Fig. 3. The standard deviation of the distributions displayed in Fig. 3 can be seen in Table 1. From a first glance, it is apparent that propagating the centerlines from the default to the varied configuration leads to a decreased variance. This holds true for all variations when comparing the standard deviations. For a differing amount of denoising iterations of the ADMIRE algorithm, the variance is relatively low in the case centerlines are propagated. For sharper kernels, mean offsets are observed ($\mu _{Bv40} =$ 0.054; $\mu _{Bv44} =$ 0.101; $\mu _{Bv49} =$ 0.094). The most considerable offset of the mean value with an amplitude of 0.167 is observed for the true stack variation with centerline propagation. When using true stack the standard deviation and therefore the variance of the prediction change is higher than with all other variations. An explanation for this behavior is that vessels at the slab edges may have the visual impression of being narrowed due to the sharp slab boundaries. A visual example of this effect is shown in Fig. 4. Analyzing different reconstruction kernels, the resulting variance in prediction seems to correlate with the sharpness of the kernel.

**Table 1:** Standard deviation of the raw prediction change for all individual variations (abbreviations as in Fig. 3) compared to the default. “Centerline (CL) Transfer” refers to the centerlines being propagated from the default to the varied configurations.

	CL transfer	Ad2	Ad4	TS	Bv40	Bv44	Bv49	Mean
Default	Without	0.220	0.225	0.355	0.243	0.233	0.307	0.226
No ensembling	Without	0.270	0.280	0.397	0.292	0.309	0.418	0.281
No TTA	Without	0.267	0.280	0.398	0.286	0.305	0.392	0.275
10% of data	Without	0.405	0.411	0.462	0.413	0.468	0.569	0.390
20% of data	Without	0.297	0.318	0.360	0.304	0.340	0.464	0.298
Default	With	0.035	0.057	0.313	0.080	0.143	0.227	0.122
No ensembling	With	0.048	0.087	0.340	0.110	0.199	0.334	0.160
No TTA	With	0.043	0.075	0.347	0.108	0.192	0.296	0.152
10% of data	With	0.030	0.077	0.286	0.148	0.261	0.433	0.176
20% of data	With	0.051	0.077	0.308	0.106	0.209	0.376	0.161

Above mentioned trends observed for the variations hold when assessing the model without ensembling, TTA, or using less training data (cf. Table 1). However, the general robustness decreases for each of these experiments compared to the default model. The highest standard deviation (± 0.390) of the prediction changes is observed when using just 10 % of the data without propagating the centerline extraction results. Results are again more robust when propagating the centerlines. However, the standard deviation still increases by at least 32% when not using TTA and up to 44% when only using 10% of the data.

**Figure 5:**
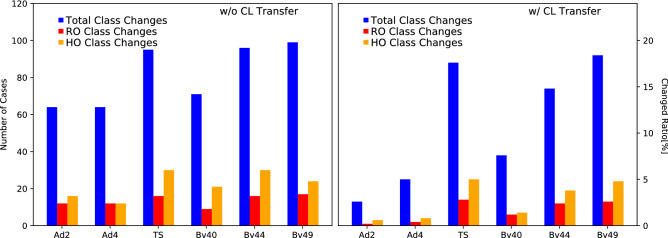
Number of samples for which the class prediction changed due to the parameter change (abbreviations as in Fig. 3). Total class changes refers to all CAD-RADS grades, RO to the rule-out and HO to the hold-out task. Note: predictions close to the thresholds easily change class bin even with small prediction changes.

### Binned prediction changes

To more directly assess the impact on the resulting clinical scores and decisions, we also show how many times the prediction changed due to the changed image formation parameters. We therefore present the number of class changes in Fig. 5. Overall, for all configuration and the full pipeline between 12 and 20% of the cases changed the predicted CAD-RADS score. A low number changes between the clinically relevant cases of rule-out and hold-out. Moreover, the same trends as described for the other metric hold true for all varied configurations.

**Figure 6:**
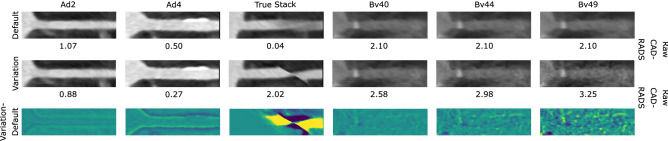
Proximal RCA segments for the cases with the largest CAD-RADS prediction deviation for each parameter configuration (note that the same patient showed the largest deviation for all possible kernel choices). Raw predictions with propagated preprocessing results are displayed for each respective configuration.

### Appearance changes

To foster intuition on why the reconstruction parameter changes lead to different predictions, we depict the stretched proximal RCA segment as fed into the NN for the cases with the respective largest CAD-RADS prediction change for each variation in Fig. 6 (all other segments are provided in the Supplementary material). When changing the ADMIRE strength to 2 or 4 the visual appearance is quite similar for a human reader, but the difference image shows slight deviations, especially around the vessel wall, which may explain the slightly different scores. For the true stack variations, the reason for the differing prediction is already apparent when looking at the deviation image: the slab boundary cuts through the vessel obscuring the image information. Lastly, we assess how the reconstruction kernel choice changes the appearance. Here we can see incrementally higher noise levels which appear slightly localized at the vessel wall as seen in the difference images.

**Figure 7:**
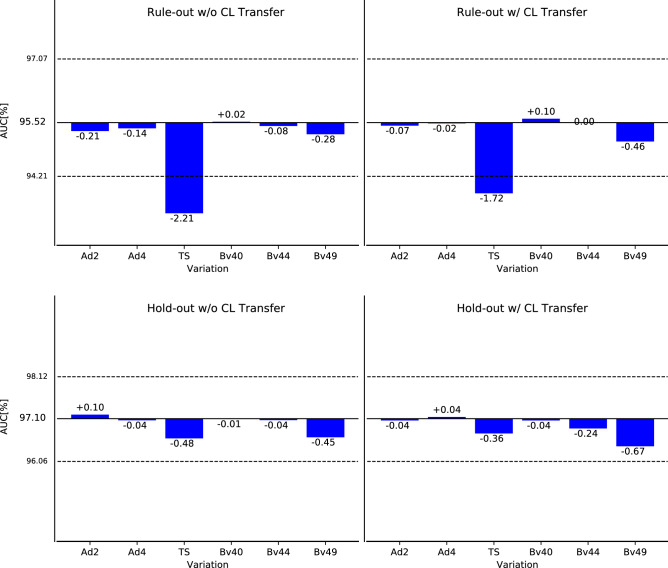
Performance on the data set A with all configurations (abbreviations as in Table 1) compared to the default for the rule-out and hold-out task, with and without centerline propagation. The dashed lines correspond to the 95% Confidence Interval (CI) for the default configuration. Note that the performance of the default configuration does not depend on the preprocessing as the centerlines of the default configuration are propagated.

**Table 2:** Performance deviation for all individual variations (abbreviations as in Fig. 3) compared to the default. “CL Transfer” refers to the centerlines being propagated from the default to the varied configurations. AUC is either displayed with 95$\%$ CI or the standard deviation over all respective single models or angles.

	Task	CL transfer	AUC [%]	Ad2	Ad4	TS	Bv40	Bv44	Bv49
Default	Rule-out	Without	95.72 [94.21, 97.07]	− 0.14	− 0.15	− 2.07	− 0.08	− 0.08	− 0.36
No ensembling	Rule-out	Without	94.65 ± 0.74	+ 0.03	− 0.15	− 1.98	+ 0.05	− 0.06	− 0.23
No TTA	Rule-out	Without	94.59 ± 0.39	− 0.09	− 0.04	− 1.58	+ 0.04	− 0.15	− 0.68
10% of Data	Rule-out	Without	86.73 [84.01, 89.52]	+ 1.25	+ 1.97	− 0.40	+ 1.29	+ 1.95	+ 2.41
20% of Data	Rule-out	Without	87.84 [85.00, 90.43]	+ 1.07	+ 0.99	− 0.69	+ 0.78	+ 1.07	+ 1.15
Default	Hold-out	Without	97.11 [96.06, 98.12]	+ 0.08	− 0.04	− 0.34	− 0.03	− 0.06	− 0.48
No ensembling	Hold-out	Without	96.79 ± 0.18	+ 0.12	− 0.13	− 0.44	− 0.06	− 0.16	− 0.62
No TTA	Hold-out	Without	96.66 ± 0.32	− 0.09	+ 0.08	− 0.42	− 0.08	− 0.30	− 0.88
10% of data	Hold-out	Without	93.01 [90.97, 94.86]	+ 0.39	+ 0.20	+ 0.08	+ 0.14	− 0.14	− 0.06
20% of data	Hold-out	Without	96.00 [94.75, 97.20]	− 0.19	+ 0.09	− 0.57	− 0.35	− 0.79	− 1.01
Default	Rule-out	With	95.72 [94.21, 97.07]	− 0.06	− 0.04	− 1.66	+ 0.06	− 0.06	− 0.54
No ensembling	Rule-out	With	94.65 ± 0.74	− 0.05	− 0.13	− 1.42	+ 0.12	+ 0.02	− 0.51
No TTA	Rule-out	With	94.59 ± 0.39	− 0.12	− 0.12	− 2.12	− 0.11	− 0.10	− 0.42
10% of data	Rule-out	With	86.73 [84.01, 89.52]	+ 0.05	+ 0.07	− 0.59	+ 0.75	+ 1.02	+ 1.24
20% of data	Rule-out	With	87.84 [85.00, 90.43]	+ 0.13	− 0.27	− 1.48	+ 0.52	+ 1.10	+ 0.92
Default	Hold-out	With	97.11 [96.06, 98.12]	− 0.05	+ 0.05	− 0.20	− 0.05	− 0.25	− 0.71
No ensembling	Hold-out	With	96.79 ± 0.18	− 0.04	+ 0.02	− 0.36	− 0.10	− 0.30	− 0.83
No TTA	Hold-out	With	96.66 ± 0.32	+ 0.07	− 0.10	− 0.48	− 0.08	− 0.17	− 0.70
10% of data	Hold-out	With	93.01 [90.97, 94.86]	+ 0.10	− 0.18	− 0.08	+ 0.07	+ 0.11	− 0.20
20% of data	Hold-out	With	96.00 [94.75, 97.20]	− 0.07	+ 0.10	− 0.83	− 0.12	− 0.33	− 0.88

### Overall performance

Besides individual prediction changes, the method’s overall performance is of interest. For the default configuration an AUC of 0.957 (95% CI [0.942, 0.971]) for the rule-out task and 0.971 (95% CI [0.961, 0.981]) for the hold-out task is achieved as displayed in Fig. 7 and in Table 2. The deviation from the results reported in Denzinger et al.^4^ is caused by the different class balance/test set evaluated. In Fig. 7 the performance deviation for the different variations is displayed as well. Interestingly, the deviation is mostly within the CI and, therefore, insignificant in these cases. The only variation leading to a significant performance drop is the use of true stack instead of mixed stacking, but only for the rule-out task. A possible explanation for this is that vessels at the stack boundaries may appear stenotic due to the sudden jump between stacks (see Fig. 4). Above observations also hold true when the centerlines are propagated from the default to the variation configurations.

Assessing the performance changes for different model configurations (no ensembling, no TTA, less training data), the findings for the default configuration hold when using no ensembling and no TTA. When training with only 10% or 20%, we can observe a significant drop in performance of our approach with larger confidence intervals, especially on the rule-out task, indicating a model that did not generalize as well. Interestingly, the performance actually improves for some of the variations. This indicates that the model focuses on different features when trained with less data and that these features are actually enhanced when the noise level varies compared to the default configuration. This reasoning at least applies to the rule-out task, where the task inherent class imbalance impacts the generalization of the model more when reducing the amount of data. Overall, the performance changes are still mostly within the 95% CIs.

## Discussion

For all evaluation metrics, it becomes apparent that the preprocessing steps have an impact on the prediction if the scan parameters are varied. The centerline extraction is likely a larger contributor regarding this behaviour as small changes in the heart isolation mask are not expected to lead to much of a difference and the centerline labeling approach only depends on the centerline points. A detailed evaluation regarding the influence of scan parameters on the centerline extraction results is not the main focus of this work, but should be subject of further research. That said, looking at the overall performance of the method, there is mostly no significant performance drop, also when considering the full pipeline. A possible explanation for this behavior is that a similar number of cases are correct for any one variation as were previously erroneous as no parameter variation causes a mean shift. However, there are two perspectives (global vs. local) regarding performance, and knowing that a slight parameter change may lead to a different diagnosis by the system for a single patient does not build trust. On the other hand, when comparing algorithms with the current gold standard—manual assessment by physicians—one must acknowledge that different readers (or one reader over time) may also grade the same or different reconstructions differently. In literature, the inter-observer variability of manual CAD-RADS scoring is reported with an inter-observer correlation (IOC) of 0.748 (average pairwise inter-observer agreement (IOA) 0.847)^19^, an IOA of 0.885^20^, or an IOC of 0.958^21^, depending on the study design and reader experience. When considering the ratio of unchanged predictions (cf. Fig. 5, right) as a metric comparable to the IOA, varying parameters like the denoising strength, and a slightly sharper reconstruction kernel are within this range. However, for the true stack configuration and even sharper kernels, the number of changed predictions increases. Looking at Fig. 1 and Fig. 6, these variations have the largest impact on the visual perception of human readers as well and may even change the perception regarding the disease state as can be seen in Fig. 4. Also, reconstruction with a sharper kernel might lead to such a high noise level that the resulting volumes are hard to read. However, no study comparing the performance of readers on the task of CAD-RADS grading for differing reconstruction kernels exist. Such a study would be hard to design as readers might be biased by their first reading under a different reconstruction configuration.

Therefore, the algorithm’s variance in prediction appears to be within the range of human readers. However, this is usually not the motivation to use algorithms as assistance tools. Algorithms are expected to yield consistent outcomes for the same patient. In particular, since the used algorithm embodiment mostly behaves as a black box, a higher robustness with respect to parameter variation is required to allow for clinical acceptance. A possible way to achieve this robustness is to include different parameter configurations into training^14^. This idea seems promising, as in the current training pipeline only reconstructions with a softer kernel are included. An additional possibility to increase robustness might be to transfer algorithms aiming to disentangle biological and technical information^16^ into the deep learning world.

Another aspect to elaborate on is whether the results reported here are transferable to other methods proposed in literature^22–30^. These mostly focus on the determination and detection of significant stenosis which is similar to the hold-out task. Usually, these approaches also rely on a prior centerline extraction usually followed by an MPR volume construction^22–27,29^. Additionally, for all approaches a CNN is used as a feature extractor. Therefore, findings presented here should be largely transferable to different architecture embodiments. Exceptions may be the works of Muscoguiri et al.^28^, who directly operate on the 3D data, and Paul et al.^30^, who operate on the curved views instead. Furthermore, all of the above-mentioned approaches were trained from data collected from a respective single site. As there usually is an internal consensus on how data is reconstructed at each individual clinical site, our choice of training our method with the data reconstructed as part of the clinical workflow is a valid and transferable choice.

Also, we evaluate whether our CAD-RADS scoring NN behaves similarly if no robustness enhancing measures like ensembling and TTA, or a smaller data collection are used. We have shown that model ensembling and TTA did not alter the findings of our study. However, a limited amount of training data leads to a less generalized model, especially regarding the rule-out task, which is most severely impacted by the task inherent class imbalance. Related works usually perform analysis on a per-vessel basis and are therefore not impacted as severely by this class imbalance. Still, the uncertainty when considering the standard deviation of the raw CAD-RADS prediction changes as a metric, behaves comparable to the default configuration.

## Conclusion

In this work, we analyzed the effects of varying image formation parameters on an existing AI-based system to automatically grade CCTA scans with the CAD-RADS score. To this end, we reconstructed 500 raw CCTA scans under eight parameter configurations, which to our knowledge are commonly applied in clinical practice. Parameter changes evaluated include the denoising strength, slab combination, and reconstruction kernel choice. We found that the preprocessing steps as well as the NN prediction step are not robust to all parameter variations. Using true stack to combine slabs of different heart phases leads to a slight overestimation of the CAD-RADS score for patients with movement between slabs as stack artifacts occurred. These artifacts can create the visual perception of a narrowed vessel at slab boundaries. We conclude that one should consider excluding datasets reconstructed with this parameter from training and application. For varied reconstruction kernels, the variance of the prediction change increased with increasing kernel sharpness. Globally, the performance remained on a high level for all variations. However, individual prediction changes occurred, which may not built trust in clinical application of such an algorithm if a patient’s scoring depends on the way their scan was reconstructed. Therefore, we conclude that strategies to create more robust predictions for individual patients need to be developed. These may include the use of a more diverse training set. However, also the preprocessing steps need some additional attention as they were contributors to the prediction changes. We have shown that the same findings hold true when leaving out robustness-enhancing measures like model ensembling and TTA. Furthermore, the method at hand behaves slightly differently when trained with less samples due to reduced generalization.

## Supplementary Information

Supplementary Information.

## Data Availability

The data are not publicly available due to data protection regulations. They are available from the authors upon reasonable request.
