# Supplementary material for: How scan parameter choice affects deep learning-based coronary artery disease assessment from computed tomography
Source: Sci Rep. 2023 Feb 13;13:2563. doi: 10.1038/s41598-023-29347-9 (PMC9925789; doi:10.1038/s41598-023-29347-9)
Supplement: Supplementary file 1 — Supplementary Information. [file 41598_2023_29347_MOESM1_ESM.pdf]

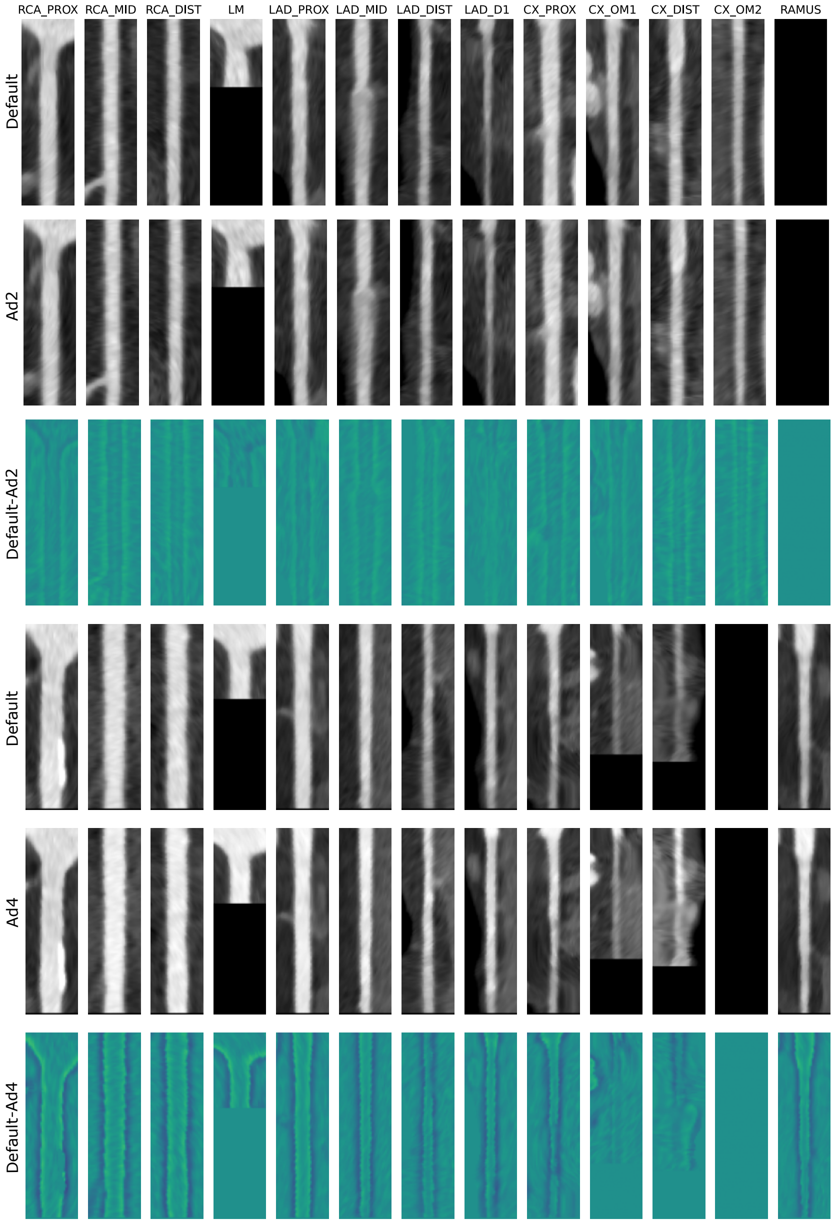

Figure 1. Appearance change due to a change in admire strength: respective case with the largest Coronary Artery Disease-Reporting and Data System (CAD-RADS) prediction deviation. Full model with propagated preprocessing results was considered. The depicted segments represent the input to the neural network. Appearance especially changes around the vessel wall, as can be seen in the difference images

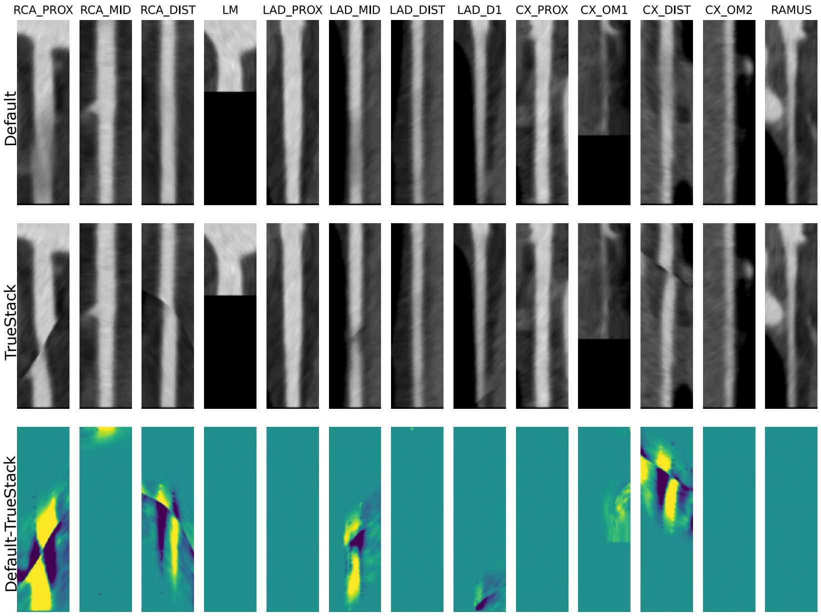

Figure 2. Appearance change due to the use of true stacking: patient with the largest CAD-RADS prediction deviation. Full model with propagated preprocessing results was considered. The depicted segments represent the input to the neural network. The sharp slab boundary cuts through several segments obscuring the image information.

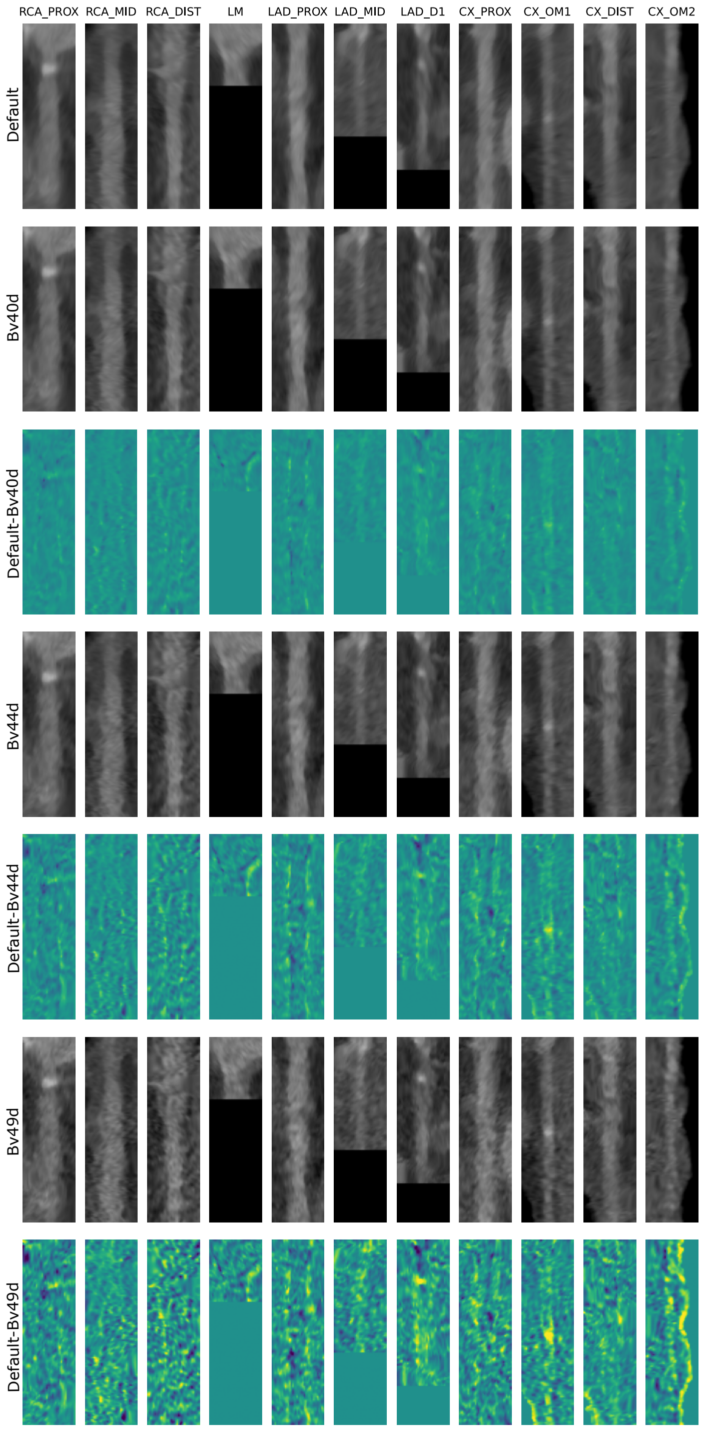

Figure 3. Appearance change due to a different reconstruction kernel: case with the largest CAD-RADS prediction deviation (Note that the same patient showed the largest deviation for all possible choices). Full model with propagated preprocessing results was considered. Non-existing segments were not visualized. Appearance changes - mainly due to more noise - increase as sharper kernels are selected.
